# Cytokines as Potential Biomarkers for Differential Diagnosis of Sepsis and Other Non-Septic Disease Conditions

**DOI:** 10.3389/fcimb.2022.901433

**Published:** 2022-06-23

**Authors:** Augustina Frimpong, Ewurama D. A. Owusu, Jones Amo Amponsah, Elizabeth Obeng-Aboagye, William van der Puije, Abena Fremaah Frempong, Kwadwo Asamoah Kusi, Michael Fokuo Ofori

**Affiliations:** ^1^ Department of Immunology, Noguchi Memorial Institute for Medical Research, College of Health Sciences, University of Ghana, Accra, Ghana; ^2^ Department of Medical Laboratory Sciences, School of Biomedical and Allied Health Sciences, College of Health Sciences, University of Ghana, Accra, Ghana; ^3^ Department of Biochemistry, Cell and Molecular Biology, College of Basic and Applied Sciences, University of Ghana, Accra, Ghana

**Keywords:** sepsis, malaria, biomarker, pro-inflammatory cytokine, anti-inflammatory cytokine, diagnosis

## Abstract

Sepsis defined as a dysregulated immune response is a major cause of morbidity in children. In sub-Saharan Africa, the clinical features of sepsis overlap with other frequent infections such as malaria, thus sepsis is usually misdiagnosed in the absence of confirmatory tests. Therefore, it becomes necessary to identify biomarkers that can be used to distinguish sepsis from other infectious diseases. We measured and compared the plasma levels of 18 cytokines (Th1 [GM-CSF, IFN-γ, TNF-α, IL-1β, 1L-2, IL-6, IL-8, IL-12/IL-23p40, IL-15], Th2[IL-4, IL-5, IL-13), Th17 [IL17A], Regulatory cytokine (IL-10) and 7 chemokines (MCP-1/CCL2, MIP-1α/CCL3, MIP-1β/CCL4, RANTES/CCL5, Eotaxin/CCL11, MIG/CXCL9 and IP-10/CXCL10 using the Human Cytokine Magnetic 25-Plex Panel in plasma samples obtained from children with sepsis, clinical malaria and other febrile conditions. Children with sepsis had significantly higher levels of IL-1β, IL-12 and IL-17A compared to febrile controls but lower levels of MIP1-β/CCL4, RANTES/CCL5 and IP10/CXCL10 when compared to children with malaria and febrile controls. Even though levels of most inflammatory responses were higher in malaria compared to sepsis, children with sepsis had a higher pro-inflammatory to anti-inflammatory ratio which seemed to be mediated by mostly monocytes. A principal component analysis and a receiver operator characteristic curve analysis, identified seven potential biomarkers; IL-1β, IL-7, IL-12, IL-1RA, RANTES/CCL5, MIP1β/CCL4 and IP10/CXCL10 that could discriminate children with sepsis from clinical malaria and other febrile conditions. The data suggests that sepsis is associated with a higher pro-inflammatory environment. These pro-inflammatory cytokines/chemokines could further be evaluated for their diagnostic potential to differentiate sepsis from malaria and other febrile conditions in areas burdened with infectious diseases.

## Background

Sepsis is a dysregulated immune response characterized by systemic inflammatory response syndrome (mild sepsis) mostly resulting from an infectious disease (Lever and Mackenzie, 2007; Chakraborty and Burns, 2019). In severe cases, it can lead to septic shock, and systemic organ dysfunction (Bone et al., 1992; Simpson, 2016). Globally, in 2017, there was an estimated 48.9 million cases of sepsis with 11 million of these resulting in death (Rudd et al., 2020). However, the incidence of the condition and its associated morbidity and mortality is highest in sub-Saharan Africa (SSA), a region plagued by various infectious diseases including malaria (Hotez and Kamath, 2009; Organization WHO, 2020; Rudd et al., 2020). Sepsis occurring in pediatrics is an important determinant of morbidity and mortality especially in SSA (Watson et al., 2003; Wiens et al., 2012; Kissoon et al., 2017). Despite the advancement in treatment and management options, accurate diagnosis of the condition is very vital to improve management and treatment outcomes.

The most common causes of sepsis include infections from bacteria, viruses and protozoans. Although infectious pathogens can cause sepsis, the condition can arise from other non-infectious diseases as well (Rudd et al., 2020). Yet, sepsis as a whole also accounts for a lot of hospital mortality of about 30%, and in patients with complications, mortality can increase to as high as 50% (Jawad et al., 2012; Kaukonen et al., 2014). Therefore, identifying biomarkers which can be used either as diagnostic or prognostic tools is of significance in disease management. Identifying potential biomarkers of sepsis will help in the management of the disease and possibly prevent severe complications such as organ dysfunction and death.

In areas like sub-Saharan Africa, the clinical features of sepsis overlaps with other frequent infections such as malaria (Bassat et al., 2009; Njim et al., 2018). Therefore, in endemic areas where these infections occur, both anti-malarial and antibiotics are co-administered (World Health Organization, 2015). However, with the impending anti-microbial resistance pandemic, it has become necessary to identify other biomarkers that can be used to distinguish sepsis from the routine symptoms of other infectious diseases. Moreover, the use of blood cultures in identifying bacteremia is very expensive, time consuming and not readily available (Nadjm et al., 2010).

The role of inflammatory mediators including cytokine and chemokine profiles, and their association with disease outcomes have been extensively studied in sepsis (Pierrakos and Vincent, 2010; Lvovschi et al., 2011; Chaudhry et al., 2013; Fjell et al., 2013). Among these, for diagnosing sepsis, host blood levels of C-reactive protein, procalcitonin and lactate are the most commonly used inflammatory mediators (Samraj et al., 2013; Ljungström et al., 2017; Ryoo et al., 2019). Nonetheless, identifying new biomarkers may help in proper disease management and in preventing unfavorable outcomes especially in children. Therefore, in this study, we aimed to identify circulating biomarkers that characterize sepsis (either promote inflammation or immune suppression), which could help differentiate children with sepsis from those with other febrile conditions, including malaria. Additionally, we tested the hypothesis that high levels of circulating inflammatory biomarkers characterize sepsis condition, and can help differentiate sepsis from other febrile conditions in resource-limited settings.

## Methods

### Ethics Statement

Ethical approval for the study was obtained from the Ethics and Protocol Review Committee of the School of Biomedical and Allied Health Sciences and the Noguchi Memorial Institute for Medical Research, College of Health Sciences, University of Ghana. Informed consent was obtained from parents/guardians of the participants before enrollment in the study. Participation in the study was voluntary and participants were free to withdraw at any point in time without penalty.

### Study Sites

The study was a combined prospective and retrospective case-control study. The study was conducted at the Princess Marie Louise (PML) Hospital in Accra and the Hohoe Municipal hospital. Clinical malaria samples used in the present study were obtained from children who were permanent residents of Hohoe in the West Semi-equatorial climatic zone of the Volta region of Ghana, located about 220 km northeast of the capital Accra. Malaria transmission intensity in this area is high and intense, with approximately 65 infectious bites per person per year (Kweku et al., 2008). *Plasmodium ovale* and *P. malariae* infection are occasionally reported, but the most dominant species of *Plasmodium* in this zone is *P. falciparum* (Kweku et al., 2008). For the purpose of this study, all samples included were from patients infected with only *P. falciparum*. Also, clinical samples from children with sepsis and febrile controls were obtained from the Princess Marie Louise hospital.

### Patient Populations

#### Malaria Diagnosis

Whole blood samples were obtained from 76 children; 33 children with clinical malaria with no sepsis, 20 children with non-malaria related fever as febrile controls and 23 children with non-malaria sepsis. Clinical malaria samples were obtained as archived samples collected under the Institutional Review Board of the Noguchi Memorial Institute for Medical Research and the Ghana Health Service. No data that allows for the identification of study participants were provided. Malaria diagnoses were performed *via* rapid diagnostic testing, using the HRP2 and LDH-based lateral flow kits as well as by Giemsa-stained blood smears, viewed by microscopy at 100x magnification under oil immersion. Additionally, microbiological cultures were performed to rule out bacterial infections. Plasma samples were obtained from the whole blood samples after centrifugation for 10 minutes at 3000 rpm and stored at -80°C until time for experiments.

#### Sepsis Diagnosis

A combination of clinical and laboratory assessments was used in sepsis diagnosis. Children diagnosed by attending physician as having sepsis presented with faster heart rate, body temperature above 38°C and chills, difficulty in breathing, and no focus on other illnesses to the hospital. Additionally, diagnostic criteria included a full blood count with thrombocytopenia and neutrophilia.

### Multiplex Immunoassay

Stored plasma samples obtained from participants were thawed on ice, and concentrations of the cytokines were determined using commercial kits and according to the kit manufacturer’s protocol. Briefly, a two-fold serial dilution of samples was prepared in 96 well plates using assay diluent whereas assay standards were reconstituted using 500µl of assay diluent. The levels of the cytokines were determined using a magnetic bead-based multiplex assay. A Human Cytokine Magnetic 25-Plex Panel (Thermo Fisher Scientific Corporation, United States) was used to estimate the levels of these immune mediators; 18 cytokines (Th1 [GM-CSF, IFN-γ, TNF-α, IL-1β, 1L-2, IL-6, IL-8, IL-12/IL-23p40, IL-15], Th2[IL-4, IL-5, IL-13), Th17 [IL17A], Regulatory cytokine(IL-10) and 7 chemokines (MCP-1/CCL2, MIP-1α/CCL3, MIP-1β/CCL4, RANTES/CCL5, Eotaxin/CCL11, MIG/CXCL9 and IP-10/CXCL10). Sample dilutions, reagents, and standards were all prepared according to the manufacturer’s protocol. The plates were read using the LUMINEX^®^ 200™ system, running on the Xponent 3.1 software. Analyte levels were reported as the concentrations.

### Statistical Analysis

Data analysis and graphs were performed using the GraphPad Prism version 9.0.0 (San Diego, CA, USA) and the R-statistical software (version 4.1.0). For data that were not normally distributed, the Kruskal-Wallis test for non-parametric testing was used with Dunn’s *post-hoc* test to correct for multiple comparisons. The associations between cytokines and chemokines were analyzed using the Spearman’s rank correlation test, whereas the corrplot package in R was used to develop the correlograms. Heatmaps were generated using the pheatmap and gplots packages in R.

Additionally, the receiver operating characteristic (ROC) curve analysis was performed to determine if any of the cytokines or chemokines measured could discriminate between children with sepsis from clinical malaria and febrile controls, giving information on their area under the curve (AUC) values as well as their specificities, sensitivities and cut-off values. Also, principal component analysis (PCA) was performed in R using the prcomp and ggplots2 functions. The PCA was performed to determine if the cytokines and chemokines selected from the ROC curve analysis could distinguish sepsis patients from malaria and febrile controls. For all statistical tests, differences with a P-value less than 0.05 was considered to be statistically significant.

## Results

### Characteristics of Study Participants

A total number of 76 children were enrolled in this study; children with clinical malaria (n=33), non-malaria sepsis (n=23) and febrile/sick controls (n = 20). The characteristics of the study participants are summarized in **Table 1**. The ages of the children were comparable (p>0.05), however, the median age of children with non- malaria sepsis was lower when compared to the other groups. The number of males enrolled did not differ significantly from the female participants.

**Table 1:** Demographics of study participants.

Characteristics	Clinical malaria	Non-malaria sepsis	Non-malaria febrile control	P value
Sample size (n)	33	23	20	NA
Sex (n)				0.85
Male	10	10	8	
Female	23	12	12	
Age (IQR) years	3.5 (2-5.75)	2.0 (1.0-4.25)	3.0 (1.28-4.0)	0.23
Parasitemia (Avg),**/**µl	94494.87	NA	NA	NA

N, number of participants; IQR, interquartile range; NA, not applicable; Avg, average.

### Pattern of CC- and CXC- Chemokines in Sepsis

**Figure 1:**
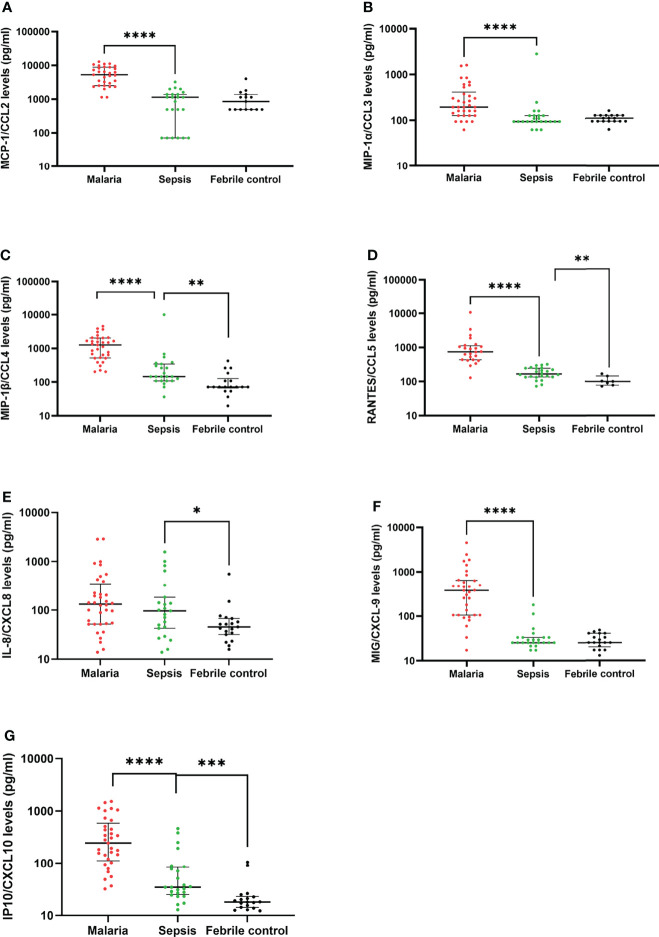
Patterns of chemokine expression in children with sepsis compared to malaria and febrile controls. The scatter plots show the plasma levels of chemokines (pg/ml) **(A-G)** detected in children with clinical malaria, sepsis and febrile controls. The plots indicate the median and interquartile ranges. Differences were considered significant when p<0.05. Statistically significant values are denoted by *p < 0.05; **p < 0.01; ***p < 0.001; ****p < 0.0001. Statistical analyses were performed using the Kruskal-Wallis with Dunn’s Multiple comparison *post-hoc* test.

Twenty-five cytokines and chemokines were quantified in plasma from the 76 study participants using a magnetic bead-based assay in order to identify those that could discriminate children with sepsis from the other groups. For the chemokines (**Figure 1**), levels of MCP-1 and MIP1-α were lower in children with sepsis compared to malaria (p<0.05), whereas comparable levels were found between children with sepsis and febrile controls (p>0.05, **Figures 1A, B**). Levels of MIP1-β/CCL4, RANTES/CCL5 and IP10/CXCL10 were significantly lower in children with sepsis when compared to children with malaria (p<0.01), but was higher when compared to febrile controls (p<0.01, **Figures 1C, D, G**). However, IL-8 (CXCL8) levels were significantly higher in children with sepsis compared to febrile controls (p<0.05), whereas comparable levels of IL-8 were found in children with sepsis and malaria (p>0.05, **Figure 1E**).

### Sepsis Is Characterized by a Higher IL-1β, IL-12 and IL-17A Response Compared to Febrile Controls

**Figure 2:**
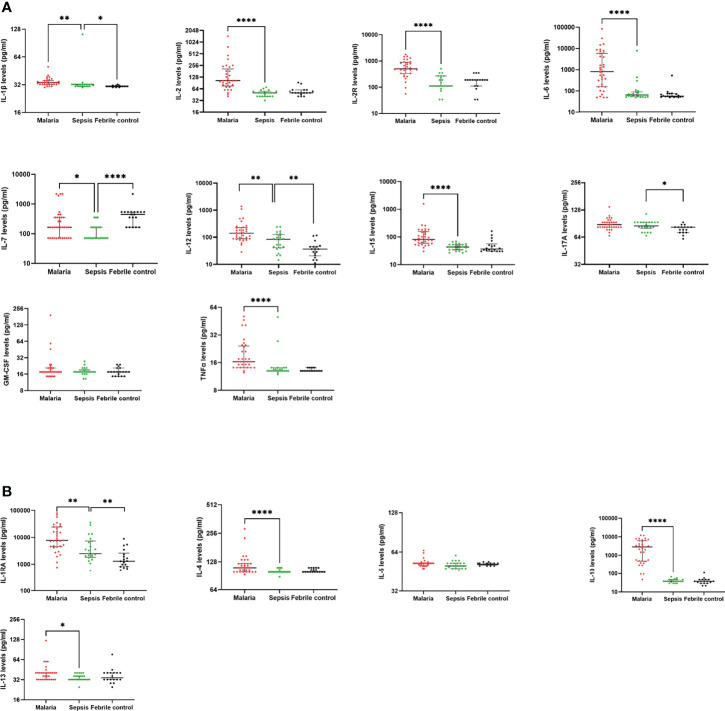
Children with sepsis show a lower anti-inflammatory profile compared to children with clinical malaria. The scatter plots show the plasma levels (pg/ml) of **(A)** pro-inflammatory cytokines, **(B)** anti-inflammatory cytokines detected in children with clinical malaria, non-malaria sepsis and febrile controls. The plots show the median and interquartile ranges. Differences were considered when p < 0.05. Statistically significant values are denoted by *p < 0.05; **p < 0.01; ****p < 0.0001. Statistical analyses were performed using the Kruskal-Wallis with Dunn’s Multiple comparison correction test.

With the aim of determining the profile of the pro-inflammatory cytokines in the non-malaria sepsis group compared to the malaria and febrile controls, levels of thirteen (13) pro-inflammatory cytokines (IL-1β, IL-2, IL-2R, IL-5, IL-6, IL-7, IL-12p40p70, IL-15, IL-17A, TNF-α, IFN-γ, IFN-α) and growth factor (GM-CSF) were determined. Levels of IFN-α and IFN-γ for patients in the sepsis and febrile control groups were mostly below the detection limit. Generally, the inflammatory status observed in children with sepsis was significantly lower, compared to children with malaria. For the pro-inflammatory cytokines, except for IL-17A and GM-CSF, which had comparable levels between the malaria and non-malaria sepsis group, significantly lower levels of pro-inflammatory cytokines were found for the children in the sepsis group. On the other hand, children with sepsis had significantly higher levels of IL-1β, IL-12 and IL-17A compared to febrile controls (**Figure 2A**), whereas only levels of IL-7 were found to be significantly lower in sepsis compared to febrile controls.

For the anti-inflammatory cytokines (IL-4, IL-5, IL-10, IL-13), only the concentrations of IL-1RA showed a significant difference between children with sepsis compared to children with either clinical malaria or febrile controls (**Figure 2B**). The median levels of IL-1RA were significantly lower in sepsis compared to malaria (p<0.01). However, when the levels in sepsis were compared to febrile controls, the concentrations were significantly increased in sepsis (p<0.01). Also, significantly lower levels of IL-4 (p<0.0001), IL-10 (p<0.0001), and IL-13 (p<0.05) were measured/detected in children with sepsis compared to children with clinical malaria. There was no significant difference in the levels of IL-5 between the groups (p>0.05).

### A High Pro-Inflammatory to Anti-Inflammatory Ratio Dominates Sepsis

Even though levels of pro-inflammatory responses were mostly higher in children with clinical malaria compared to children with non-malaria sepsis, we went on to further determine if immune responses during sepsis was dominated by a pro-inflammatory or anti-inflammatory response. Therefore, the ratio of IL1β, IL2, IL2R, IL6, IL7, IL12, IL17A and TNF α to IL10 were analyzed. It was observed that the ratio of pro-inflammatory to anti-inflammatory cytokine concentrations were significantly higher in children with sepsis compared to children with clinical malaria for all the cytokine ratios analyzed (p<0.0001, **Supplementary Figure 1**).

### Inflammatory Status in Sepsis Is Mediated Mostly by Monocyte Derived Cytokines

**Figure 3:**
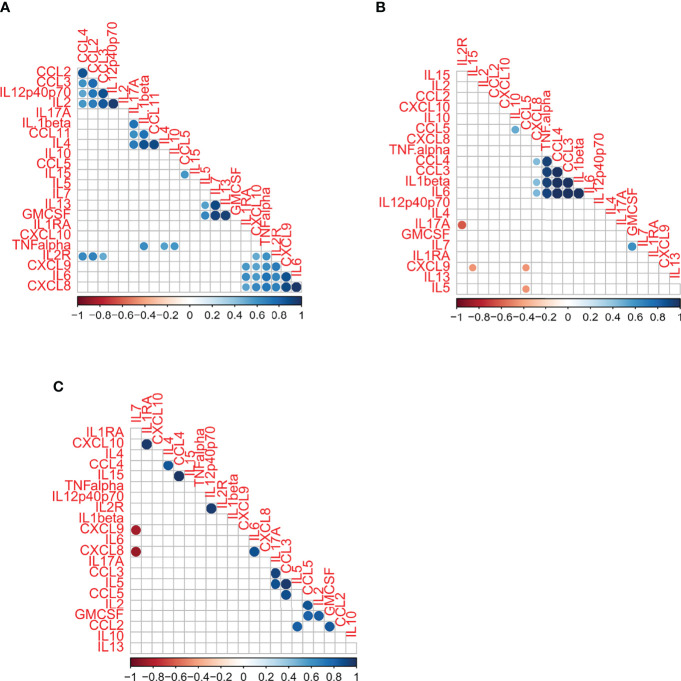
A correlation matrix/correlogram describing distinct patterns of immunological interactions of cytokines in children with **(A)** Clinical malaria **(B)** Sepsis and **(C)** Febrile controls. The matrix displays a Spearman’s rank correlation coefficient with significant association (p < 0.05) depicted by a coloured dot/circle. Non-significant correlations are depicted by empty squares. The strength of the association is indicated by the colour schemes ranging from -1 to 1 in each figure.

Both CCL3 and CCL4 are macrophage inflammatory proteins (MIP) which can further stimulate monocytes to produce other pro-inflammatory cytokines such as IL-1β, IL-6 and TNFα (Arango Duque and Descoteaux, 2014; Sindhu et al., 2019). We performed a Spearman’s correlation analysis (r) to ascertain the cytokine interactions among the sepsis, malaria and febrile control groups (**Figure 3**). There was a clustering of CXCL8, TNFα, CCL4, CCL3, IL-1β and IL-6in the sepsis group indicating a chronic inflammatory response which seems to be mediated mostly by monocytes, whereas the febrile controls had no specific pattern. However, for the malaria group, four distinct correlation patterns were observed; first pattern (CCL4, CCL2, CCL3, IL12 and IL2), second pattern (IL-17A, IL-1β, CCL11, IL4), third pattern (IL5, IL7, IL13), and fourth pattern (IL1RA, CXCL10, TNFα, IL2R, CXCL9, IL6). Importantly, three out of the four patterns for malaria had anti-inflammatory response cytokines in the clusters (IL-4, IL-13, IL-1RA). Also, although IL-10 was not in any of the four patterns for malaria, there was a positive correlation between IL-10 and TNFα levels.

From **Figure 2**, it could be observed that the cytokine and chemokines in children with sepsis had less interactions compared to children with malaria. Also, whereas both significant positive and negative interactions were observed in both the sepsis and febrile groups, only significant positive interactions were observed in the malaria group. The negative interactions for the sepsis group were between CCL5 and CXCL9, CCL5 and IL-5, and IL2R and IL-17A. Additionally, compared to responses in sepsis, malaria seems to be mediated by both adaptive and innate immune responses.

### The Potential of IL-1β, IL-7, IL-12, IL-1RA, and RANTES/CCL5, MIP1β/CCL4, IP10/CXCL10 to Differentiate Children With Sepsis From Malaria and Febrile Controls

**Figure 4:**
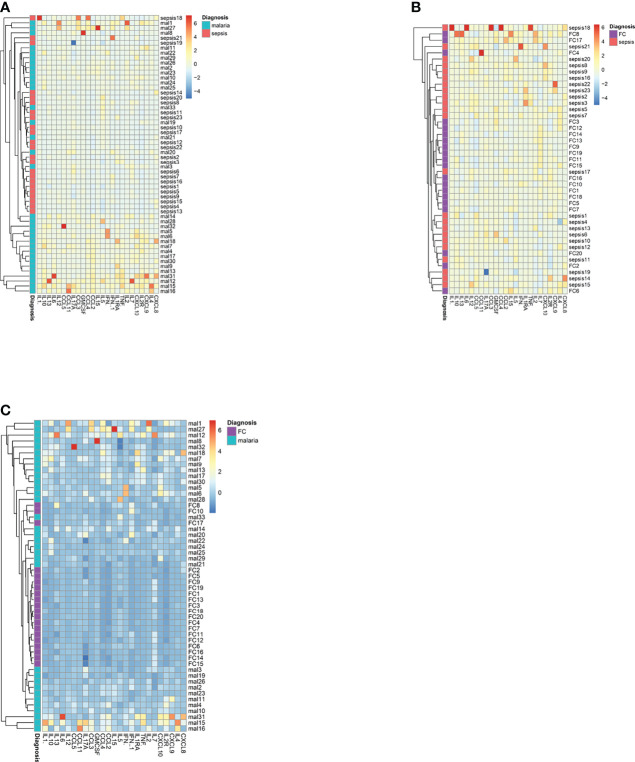
A heatmap of the plasma levels of cytokines and chemokines in the patients with sepsis, malaria and febrile controls. The heatmaps depict the ability to separate **(A)** sepsis patients from malaria patients; **(B)** sepsis patients from febrile controls and **(C)** febrile controls form malaria patients.

Next, we used a heatmap with hierarchical clustering analysis for the plasma levels of immune molecules to determine whether there may be patterns in the anti-inflammatory or inflammatory responses that may result in clustering of the sepsis group from the clinical malaria or febrile control group (**Figure 4**). We found that compared to the sepsis group, better segregation of the diseased states was found between malaria and febrile controls (**Figure 4C**). The segregation of sepsis from malaria and sepsis and febrile controls were observed to be similar.

We therefore performed a ROC analysis to determine if the levels of any of the cytokines or chemokines can be used to differentiate between sepsis and malaria as well as sepsis and febrile controls. The area under the curve (AUC), the sensitivity and specificity values, the cut-off values and significance are shown in **Table 2**.

**Table 2:** List of immunological molecules and their potential to differentiate children with sepsis from clinical malaria and febrile controls.

Cytokines	AUC	Clinical malaria				Febrile controls				P-value			Sensitivity	Specificity	Cut off (pg/ml)	P-value	AUC	Sensitivity	Specificity	Cut off (pg/ml)	
IL-1β	0.78	0.91	0.64	<32.6	***	0.77	0.65	0.78	>31.09	**
IL-1RA	0.75	0.52	0.88	<2623	**	0.76	0.83	0.67	>1717	**
IL-7	0.69	0.61	0.67	<118.6	*	0.92	0.61	1.00	>118.6	****
IL-12	0.74	0.61	0.79	<88.5	**	0.75	0.65	0.78	>46.7	**
										
Chemokines	AUC	Sensitivity	Specificity	Cut off (pg/ml)	P-value	AUC	Sensitivity	Specificity	Cut off (pg/ml)	P-value
CCL4	0.9	0.7	0.91	<282.9	****	0.79	0.91	0.67	>81	***
CCL5	0.96	0.88	0.96	<275.5	****	0.82	0.92	0.71	>103.7	**
CXCL10	0.86	0.75	0.85	<77.8	****	0.82	0.75	0.89	>26.51	***

*p < 0.05; **p < 0.01; ***p < 0.001; ****p < 0.0001.

In comparing the ROC for the various molecules, we identified four (4) cytokines (IL-1β, IL-7, IL-12 IL-1RA) and three (3) chemokines (RANTES/CCL5, MIP1β/CCL4, IP10/CXCL10) that were statistically significant and could at the same time differentiate between children with sepsis and clinical malaria and children with sepsis from other febrile controls (**Table 2**). Among the cytokines identified, IL-1β had the highest AUC value (0.78; p <0.001) in differentiating children with sepsis from clinical malaria, whereas IL-7 showed a greater potential in distinguishing patients with sepsis from febrile controls (AUC of 0.92, p<0.0001).

The chemokines had higher AUC values in differentiating between children with malaria and sepsis (p<0.0001), with CCL5 showing the greatest potential with an AUC value of 0.96. For distinguishing between sepsis from febrile controls, both CCL5 (0.82, p<0.01) and CXCL10 (0.82, p<0.001) showed the greatest potential in differentiating the two groups.

**Figure 5:**
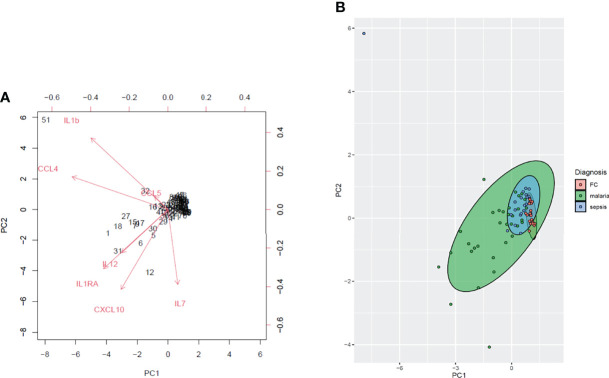
A principal component analysis (PCA) of the plasma cytokines and chemokines measured in the sepsis (n=23), malaria (n=32) and febrile controls (n=30). **(A)** The biplot depicts the principal components of the immune parameters. The PCA plot shows the levels of the measured plasma cytokines and chemokines (IL-1β, IL-7, IL-12, IL-1RA, and RANTES/CCL5, MIP1B/CCL4, IP10/CXCL10). The angles between the variables indicate the level of correlation whilst the lengths of the arrows show the variance. **(B)** The green, blue and red symbols represent the malaria, sepsis and febrile controls respectively whereas the corresponding ellipses represent the clusters of the various populations respectively.

In order to visualize the patterns and variations in the dataset we performed a two-dimensional model principal component analysis (PCA) (**Figure 5**). Using these seven parameters, we found that the model could explain 51.5% (PC1 = 39.3; PC2 = 15.8) of the variance in the data. The PCA showed distinct profiles for the immune parameters.

## Discussion

Cytokines, chemokines and growth factors have a systemic effect during host response to an infection, injury or immunological insult. Cytokines and chemokines are also gaining popularity in the development of point-of-care diagnostics for inflammation mediated disease (Liu et al., 2021). In this study, we aimed to identify immunological molecules that could characterize sepsis and help distinguish sepsis from other febrile conditions including clinical malaria. We observed that despite most cytokine and chemokine levels being lower in sepsis when compared to clinical malaria, sepsis was dominated by a significantly higher pro-inflammatory to anti-inflammatory ratio compared to malaria. Additionally, using an ROC curve analysis, the plasma levels of IL-1β, IL-7, IL-12, IL-1RA, RANTES/CCL5, MIP1B/CCL4 and IP10/CXCL10 could help differentiate children with sepsis from both clinical malaria and febrile controls. Furthermore, using an unsupervised learning algorithm (PCA), we observed that levels of these seven mediators could indicate distinct patterns in the study groups.

Chemokines are small immunological molecules that function to recruit and activate immune cells including leukocytes to the site of infection or invasion. Among the CCL- and CXCL- chemokines measured in this study, CCL4 and CCL5, as well as CXCL-10 were found to be significantly different among all the three study groups. Both CCL4 and CCL5 are inflammatory chemokines involved in the chemotaxis of leucocytes, including monocytes and lymphocytes. Importantly, the levels of CCL4, CCL5 and CXCL10 have been reported to be increased in both sepsis and malaria (Cavaillon et al., 2003; Ioannidis et al., 2014; Dunst et al., 2017). While increased levels of these chemokines have also been associated with poor clinical outcomes (Ioannidis et al., 2014), chemokines like CCL5 and CCL4 have been known to change the inflammatory environment towards wound healing and repair (Gillitzer and Goebeler, 2001; Ridiandries et al., 2018). The increased levels of these chemokines in malaria and sepsis compared to the febrile controls supports the notion that these two diseases are associated with cellular activation which can lead to tissue damage.

Even though most of the pro-inflammatory cytokines in sepsis were lower than those recorded in malaria, levels of CXCL8/IL-8 and IL-17 were comparable between the malaria and sepsis group. IL-8 is an inflammatory mediator produced predominantly by macrophages and monocytes needed for neutrophil chemotaxis whereas IL-17A is a potent mediator which increases chemokine production in various tissues for monocyte recruitment (Baggiolini et al., 1989; Kovach and Standiford, 2012; Chaudhry et al., 2013; Aitken et al., 2018). Importantly, none of these inflammatory mediators could discriminate between sepsis and clinical malaria. This may imply that both sepsis and malaria are mediated by acute and chronic inflammatory mediators.

IL-1Ra, the antagonist to IL-1β, has been considered a novel treatment against sepsis since it inhibits excessive inflammation (van Deuren et al., 1994). Interestingly, IL-1Ra was the only anti-inflammatory cytokine that showed significant difference between children with sepsis from those with malaria and the febrile controls. Generally, the initial phase of sepsis is characterized by an acute inflammation at the site of invasion which later proceeds to systemic inflammation leading to the recruitment and activation of immune cells; then the immunosuppressive stage where anti-inflammatory cytokines such as IL-1Ra are released. Even though IL-1Ra has been reported to reduce excessive inflammation, an IL-1Ra mediated immunosuppressive environment may lead to poor disease outcome in sepsis, whereas in malaria this has been associated with high parasitemia (Gogos et al., 2000; Oyegue-Liabagui et al., 2017).

Also, IL-7 levels in sepsis were significantly lower compared to the malaria and febrile control groups. Its high levels in the malaria group are expected since the paroxysmal fever which is associated with *P. falciparum* infection is triggered by strong pro-inflammatory responses. Lower levels of IL-7 in the sepsis group may be due to its important role in reversing the immunosuppressive state in sepsis (Bosmann and Ward, 2013). IL-7 is known to prevent apoptosis during sepsis by inducing increased expression of the anti-apoptotic protein Bc12. Additionally, IL-7 has gained the interest in recent times as it has been found to have both diagnostic and therapeutic potentials in sepsis cases (Mackall et al., 2011; Francois et al., 2018).

It has been observed that the systemic inflammatory response syndrome may result from an imbalance between the pro-and anti-inflammatory immune responses. Normally, the production of pro-inflammatory cytokines is counteracted by the release of anti-inflammatory mediators such as IL-10 to prevent tissue damage. However, the release of counter-inflammatory response may be time dependent and could be affected by time of sampling as well as baseline immune status of the host (Blackwell and Christman, 1996; Gogos et al., 2000). Similarly, in sepsis, there is a mutual relationship between the pro- and anti-inflammatory mediators that contribute to disease pathogenesis (Xiao et al., 2011; Iskander et al., 2013; Matsumoto et al., 2018), and a balance between these have been associated with good clinical outcomes. From our studies we observed that children with sepsis had a dysregulated immune response dominated by a higher pro-inflammatory environment which could contribute to disease manifestation. Indeed, it would have been interesting to determine if sampling at different time points may provide a shift towards an anti-inflammatory response. The correlation matrices in the sepsis group produced a single cluster showing a positive interaction among the pro-inflammatory mediators (CCL3, CCL4, IL1β, IL-6 and TNFα). It is well documented that TNFα, IL-6 and IL1β produced by macrophages promote Th1 cells development (Arango Duque and Descoteaux, 2014). This may indicate that the initial phase of sepsis, characterized by acute inflammation, may be macrophage driven. Besides, the immuno-metabolism role of macrophages in sepsis has been considered to be crucial and targeted for therapeutic approaches (Kaukonen et al., 2014).

Other studies have identified the use of IL-1β, CCL4, CCL5 and CXCL10 as possible diagnostic markers for sepsis (Ng et al., 2007; Kurt et al., 2007). Here, using the principal component analysis and the receiver operator characteristic curve analysis, we identified seven potential markers; three pro-inflammatory cytokines IL-1β, IL-7, IL-12; the anti-inflammatory cytokine IL-1RA, and the pro-inflammatory chemokines RANTES/CCL5, MIP1β/CCL4, IP10/CXCL10 that could discriminate between children with sepsis from those with clinical malaria and other febrile conditions. Furthermore, performing a principal component analysis with these seven biomarkers could segregate the sepsis from the malaria and febrile controls. Therefore, these biomarkers should further be investigated in larger cohorts to inform clinical decisions.

In summary, the data suggests that sepsis is associated with a dysregulated immune response characterized by a higher pro-inflammatory environment. The high inflammatory response may be related to the progression of disease leading to tissue and organ damage. Additionally, these pro-inflammatory cytokines and chemokines could further be evaluated for point-of-care diagnostic potential to differentiate between malaria and other febrile conditions in areas burdened with infectious diseases such as sub-Saharan Africa.

## Data Availability Statement

The raw data supporting the conclusions of this article will be made available by the authors, without undue reservation.

## Ethics Statement

The studies involving human participants were reviewed and approved by Institutional Review Board of the Noguchi Memorial Institute for Medical Research and the Ethics and Protocol Review Committee of the School of Biomedical and Allied Health Sciences, University of Ghana. Written informed consent to participate in this study was provided by the participants’ legal guardian/next of kin.

## Author Contributions

AF, EO and MO conceived the idea and designed the experiments. AF, EO and MFO supervised the work. JA, EO and AFF performed the experiments in the study. AF, EO, WV, KK and MO wrote the paper. All authors read and approved the final manuscript.

## Conflict of Interest

The authors declare that the research was conducted in the absence of any commercial or financial relationships that could be construed as a potential conflict of interest.

## Publisher’s Note

All claims expressed in this article are solely those of the authors and do not necessarily represent those of their affiliated organizations, or those of the publisher, the editors and the reviewers. Any product that may be evaluated in this article, or claim that may be made by its manufacturer, is not guaranteed or endorsed by the publisher.

## Supplementary Material

Supplementary Figure 1 Increased pro-inflammatory/anti-inflammatory cytokine ratios in children with sepsis compared to clinical malaria. The scatter plots **(A–E)** show the ratios of the pro-inflammatory/anti-inflammatory cytokines (IL-2/IL10, IL-6/IL10, IL-7/IL-10, IL-12/IL-10, IL-17A/IL-10 and TNF-α/IL-10) estimated in children with sepsis and clinical malaria. The plots indicate the median and interquartile ranges. Significant differences were considered when p<0.05. Statistically significant values are denoted as ****p < 0.0001. Statistical analyses were performed using the Mann Whitney U test.
