# Supplementary figures and images for: Cytokines as Potential Biomarkers for Differential Diagnosis of Sepsis and Other Non-Septic Disease Conditions
Source: Front Cell Infect Microbiol. 2022 Jun 23;12:901433. doi: 10.3389/fcimb.2022.901433 (PMC9260692; doi:10.3389/fcimb.2022.901433)

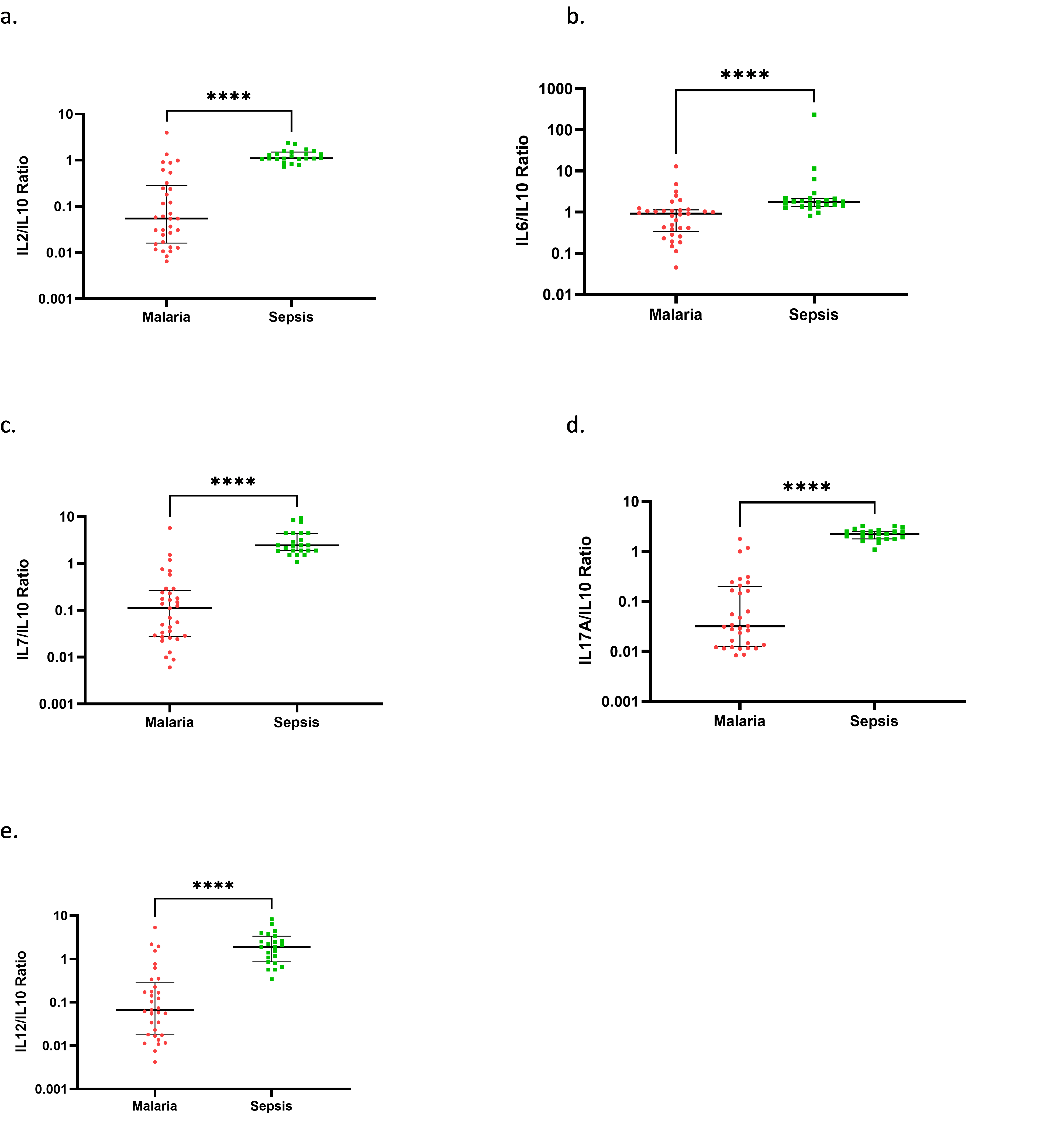

Supplement: Supplementary Figure 1 — Increased pro-inflammatory/anti-inflammatory cytokine ratios in children with sepsis compared to clinical malaria. The scatter plots (A–E) show the ratios of the pro-inflammatory/anti-inflammatory cytokines (IL-2/IL10, IL-6/IL10, IL-7/IL-10, IL-12/IL-10, IL-17A/IL-10 and TNF-α/IL-10) estimated in children with sepsis and clinical malaria. The plots indicate the median and interquartile ranges. Significant differences were considered when p<0.05. Statistically significant values are denoted as ****p < 0.0001. Statistical analyses were performed using the Mann Whitney U test. [file Image_1.tif]
